# Active Range of Motion in Non-Impingement Directions After Hip Arthroscopy for Femoroacetabular Impingement Syndrome

**DOI:** 10.3390/jcm15114313

**Published:** 2026-06-02

**Authors:** Łukasz Stołowski, Gino Kerkhoffs, Tomasz Piontek

**Affiliations:** 1 Department of Orthopedic Surgery, Rehasport Clinic, 60-201 Poznan, Poland; 2 Doctoral School, Poznan University of Medical Sciences, 60-812 Poznan, Poland; 3 Department of Orthopedic Surgery, Amsterdam UMC, Academic Medical Center, Meibergdreeg 9, 1105 Amsterdam, The Netherlands; g.m.kerkhoffs@amsterdamumc.nl; 4 Department of Spine Disorders and Pediatric Orthopedics, University of Medical Sciences Poznan, 61-701 Poznan, Poland

**Keywords:** femoroacetabular impingement syndrome, hip arthroscopy, range of motion, inertial measurement units, IMU sensors, hip biomechanics, HOOS

## Abstract

**Background**: Femoroacetabular impingement syndrome (FAIS) is a common cause of hip pain and functional limitation in young and physically active individuals. Although hip arthroscopy is an established treatment when conservative management fails, objective data on early postoperative changes in active hip range of motion (ROM) remain limited. This study aimed to evaluate changes in active hip ROM three months after arthroscopic treatment for FAIS using inertial measurement units (IMUs) and to investigate their relationship with patient-reported outcomes. **Methods**: A prospective cohort of forty-two patients (mean age 36 ± 9 years; 64% male) undergoing hip arthroscopy for FAIS was assessed preoperatively and at a three-month follow-up. Active hip ROM—including flexion, internal rotation, external rotation, and total rotation—was measured using IMU sensors, while subjective outcomes were evaluated using the Hip disability and Osteoarthritis Outcome Score (HOOS). **Results**: Significant improvements were observed across all HOOS subscales at follow-up (all *p* < 0.001). The HOOS Total score increased from 57 ± 15 to 83 ± 11. Clinical recovery, defined by the Minimal Important Change (MIC), was achieved by 74% of patients for Symptoms, 81% for Pain, 81% for ADL, 83% for Sport, and 67% for Quality of Life (QOL). Active hip ROM in the operated hip increased significantly for internal rotation (19.6° ± 11.9° to 26° ± 8°), external rotation (37° ± 10° to 40° ± 8°), and total rotation (57° ± 15° to 67° ± 12°). Changes in hip flexion were not clinically meaningful (98° to 100°), and no changes were observed in the non-operated hip. Spearman’s analysis showed weak and inconsistent correlations between active ROM and HOOSs (r ranging from −0.34 to 0.31). **Conclusions**: Hip arthroscopy for FAIS leads to early improvements in both patient-reported outcomes and active hip mobility, particularly in rotational movements, although the relationship between ROM and subjective outcomes appears weak.

## 1. Introduction

Femoroacetabular impingement syndrome (FAIS) is a leading cause of hip pain and functional limitation in young, active individuals. According to current clinical standards, FAIS is defined as a clinical triad of symptoms—including pain, stiffness, and restricted range of motion (ROM)—associated with structural conflict arising from morphological variations in the proximal femur (CAM) and/or acetabulum (Pincer) [1]. This condition frequently leads to progressive damage at the chondrolabral junction and is recognized as a primary risk factor for the premature development of hip osteoarthritis [2].

In most clinical scenarios, conservative management—comprising activity modification, targeted physical therapy, and intra-articular injections—is the first-line approach. However, if symptoms persist, arthroscopic intervention is the recommended treatment [2]. This procedure typically involves the correction of the impingement morphology (osteoplasty) and the repair or reconstruction of the acetabular labrum and cartilage. A critical, yet often overlooked, aspect of the surgical approach is capsulotomy, which is necessary for joint distraction and visualization. While the capsule is typically sutured at the end of the procedure, the integrity and healing of the capsulolabral complex significantly influence joint stability and early postoperative biomechanics [3].

The efficacy of hip arthroscopy is traditionally evaluated through a combination of subjective patient-reported outcome measures (PROMs) and objective clinical assessments. PROMs, such as those validated by Kemp et al., provide essential data on the patient’s perception of pain and quality of life [4]. Objectively, successful arthroscopic treatment has been shown to improve strength, functional performance, and ROM [5,6,7]. A recent meta-analysis highlighted significant improvements in internal rotation and flexion following surgery for FAIS [8].

Despite these findings, a significant gap remains in the literature. Most studies focus exclusively on passive ROM or measurements within the primary “impingement zones” (e.g., flexion and internal rotation). However, emerging evidence suggests that FAIS patients also exhibit movement restrictions in non-impingement directions, indicating a more global dysfunction of the hip joint complex [9,10]. Additionally, there is a paucity of studies directly comparing objective functional outcomes with patient-reported measures following hip arthroscopy [11,12].

Furthermore, traditional goniometric measurements often lack the precision and dynamic capability required to capture subtle changes in active movement during the early recovery phase. Inertial Measurement Units (IMUs) offer a solution to these limitations. Unlike traditional motion analysis or manual goniometry, IMUs provide a portable, high-precision, and objective means of assessing dynamic, active ROM in a clinical setting. A previous study demonstrated that IMUs are a feasible and reliable tool for evaluating hip kinematics, revealing significant limitations in active flexion and total rotation in symptomatic FAIS patients compared to healthy controls [13]. The present study builds directly upon those findings by examining the same cohort three months after arthroscopic treatment. This early postoperative milestone is critical, as it coincides with the completion of initial tissue healing and the transition to functional loading. The aim of this work was to quantify changes in active ROM using IMU technology and to explore the correlations between these objective biomechanical parameters and subjective functional outcomes as measured by the Polish version of the Hip disability and Osteoarthritis Outcome Score (HOOS) questionnaire. It was hypothesized that arthroscopic treatment for FAIS would lead to a significant increase in active hip ROM, particularly in rotational movements, and that these objective biomechanical improvements would positively correlate with improvements in patient-reported clinical outcomes.

## 2. Materials and Methods

### 2.1. Participants

The present study represents a 3-month follow-up analysis of a previously recruited prospective cohort of patients undergoing hip arthroscopy for symptomatic FAIS.

Initially, 53 patients were enrolled between July 2022 and August 2024. Eligibility for surgery was determined by a single experienced orthopedic surgeon (25 years of practice) based on medical history, clinical examination, and relevant imaging findings. FAIS was defined according to the Warwick Agreement as a clinical triad comprising symptoms, positive clinical signs (such as restricted ROM), and diagnostic imaging findings showing cam and/or pincer morphology [1]. Intraoperative assessment was used to confirm the diagnosis and to identify concomitant intra-articular pathologies, including acetabular labrum tears and chondral lesions, which are recognized as integral features of the clinical presentation of FAIS. All such lesions were addressed during the surgical procedure to restore joint integrity.

Inclusion criteria were age 18–60 years and referral for hip arthroscopy due to FAIS symptoms refractory to at least 12 weeks of conservative management. Exclusion criteria comprised other musculoskeletal or systemic conditions that could potentially affect the study outcomes.

**Figure 1:**
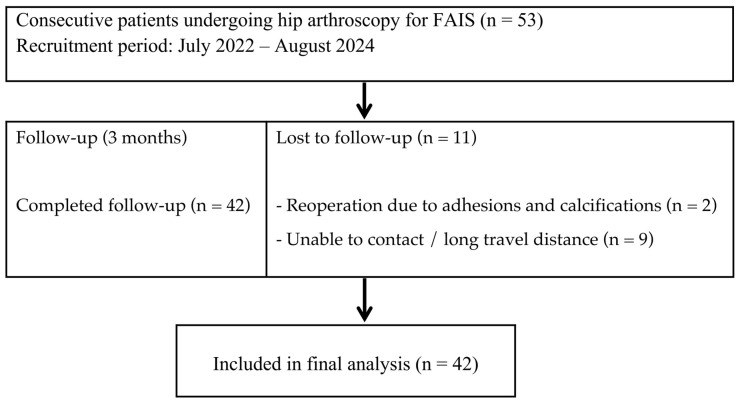
Flow diagram of patient inclusion and 3-month follow-up.

At the 3-month follow-up, 42 patients completed the full assessment and were included in the final analysis. Eleven patients were excluded from the follow-up analysis: two underwent reoperation before the 3-month evaluation due to adhesions and calcifications, and nine were lost to follow-up (inability to contact or long travel distance) (Figure 1).

The study was approved by the Bioethical Committee of the University of Medical Sciences in Poznań (no. 13/21, 14 January 2021) and all participants provided written informed consent.

### 2.2. Surgical Procedure

All hip arthroscopies were performed in a single specialized orthopedic clinic with a focus on sports-related injuries. All procedures were carried out by a single surgeon with over 25 years of overall surgical experience and more than 15 years of specific experience in hip arthroscopy. Patients were positioned supine on the operating table, and the affected hip was placed under traction. While the number and location of portals were tailored to each patient’s specific anatomy and pathology, the standard anterolateral and midanterior portals were utilized in most cases. Intraoperative data collected included the type of intra-articular pathology and the procedures performed. Fluoroscopy was used to guide portal placement and to confirm adequate cam and/or pincer resection. A limited capsulotomy was performed in all cases, followed by routine capsular repair.

### 2.3. Postoperative Rehabilitation

Postoperative rehabilitation was conducted according to the four-phase protocol described in a previous work [14]. The program was based on functional rather than time-dependent criteria and was individualized according to each patient’s goals, physical capacity, and rate of recovery. During the initial phase (0–2 weeks), the primary objectives were to protect the operated structures, reduce pain and swelling, and prevent adhesions and muscle inhibition. ROM was initially restricted to 0° of extension, 30° of abduction, 20° of external rotation, and 90° of flexion. Patients ambulated with two crutches using a foot-flat weight-bearing technique according to the surgeon’s recommendations. In the subsequent phase (approximately 2–6 weeks), rehabilitation focused on gait re-education, gradual restoration of hip mobility, and the introduction of light strengthening exercises. Between 4 and 12 weeks postoperatively, the program emphasized restoring full ROM, muscle strength, and endurance comparable to the contralateral limb. After 12 weeks, patients progressed to functional and sport-specific training aimed at regaining full performance and safe return to unrestricted activity. All participants were educated about the rehabilitation protocol and its functional progression criteria, even if therapy was conducted outside the clinic, to ensure consistency with the standardized rehabilitation framework.

### 2.4. Procedure

Participants were assessed at two time points: preoperatively and at the 3-month postoperative follow-up. Demographic data, patient-reported outcomes (HOOS), and active ROM measured using IMU sensors (RSQ Motion, RSQ Technologies, Poznań, Poland) were collected. Radiological data and intraoperative findings were obtained from the patient database.

Active ROM assessment followed the previously validated protocol, including standing flexion and prone internal/external rotation tests with sensor placement and stabilization as previously described [13]. Patient-reported outcomes were evaluated using the HOOS, which assesses symptoms, pain, activities of daily living, sports, and quality of life on a 0–100 scale, with higher scores indicating better outcomes [15].

### 2.5. Statistical Analysis

Categorical variables were described using means and standard deviations (SD), while qualitative variables were presented as frequencies (n) and percentages (%). The normality of data distribution was assessed using the Shapiro–Wilk test. To compare changes between the two time points (Preoperation and at 3 months), the Wilcoxon signed-rank test for dependent samples was employed.

To quantify the magnitude of differences for non-parametric data, effect sizes were calculated using Cohen’s r coefficient (r = Z/√N), with values of approximately 0.10–0.29 interpreted as small, 0.30–0.49 as moderate, and ≥0.50 as large effects. For parametric comparisons, including active ROM analysis, Cohen’s d was utilized, with values of approximately 0.20–0.49 considered small, 0.50–0.79 moderate, and ≥0.80 large effects.

Clinical changes in patient outcomes were evaluated using the Minimal Important Change (MIC) for individual HOOS subscales, based on established psychometric thresholds for hip arthroscopy by Kemp et al. [4]. The following thresholds were applied to identify clinical responders: 9 points for Symptoms, Pain, and Quality of Life (QOL); 6 points for ADL; and 10 points for Sport. The proportion of participants achieving the MIC was calculated as the percentage of individuals whose change met or exceeded these thresholds.

Changes in active ROM between the operated and non-operated limb across the two time points were analyzed using a two-way repeated-measures analysis of variance (2 × 2 ANOVA). In cases of significant interactions, Tukey’s HSD post hoc test was performed. The relationship between selected parameters was examined using the non-parametric Spearman’s rank correlation coefficient. All statistical analyses were performed using Statistica software (version 13.0, StatSoft, Tulsa, OK, USA). The level of statistical significance was set at *p* ≤ 0.05.

Generative AI-assisted technologies ChatGPT (OpenAI, GPT-5.3-mini, San Francisco, CA, USA), Gemini (Google LLC, Gemini 1.5, Mountain View, CA, USA), Grammarly (Grammarly Inc., San Francisco, CA, USA) were used to improve the linguistic quality and clarity of the manuscript. The authors critically reviewed and edited all AI-assisted content and take full responsibility for the final version of the manuscript.

## 3. Results

### 3.1. General Characteristics of Participants

The study included 42 patients who underwent hip arthroscopy for FAIS. The baseline demographic and clinical characteristics of the participants are presented in Table 1.

### 3.2. Hip Disability and Osteoarthritis Outcome Score (HOOS)

Significant improvements were observed in the HOOS Total score and all HOOS subscales at the 3-month follow-up compared with baseline (Table 2). The HOOS Total score increased by a mean of 26 points (*p* < 0.01; large effect size).

Based on the MIC thresholds, the following clinical improvements were observed: 31 (74%) participants reached the MIC for HOOS Symptoms (mean increase of 22 points; *p* < 0.01), and 34 (81%) patients achieved the MIC for HOOS Pain (mean increase of 27 points; *p* < 0.01). Regarding HOOS ADL, a mean increase of 26 points was recorded (*p* < 0.01), with 34 (81%) participants reaching the MIC. The HOOS Sport subscale showed the largest mean increase of 33 points (*p* < 0.01; large effect size), with 35 (83%) participants achieving the MIC. Finally, HOOS QOL improved by an average of 26 points (*p* < 0.01), and 28 (67%) participants met the MIC.

### 3.3. Active ROM

As shown in Table 3, significant time × side interactions were observed for hip external rotation, internal rotation, and total rotation, indicating side-specific improvements in the operated hip over the 3-month follow-up. No significant interaction effect was found for hip flexion.

The rotational parameters of the operated hip demonstrated robust clinical improvements characterized by substantial effect sizes. External rotation increased with a moderate-to-large effect size (Cohen’s d ≈ 0.7), while internal rotation demonstrated a large effect size (d ≈ 1.2), reflecting substantial restoration of transverse-plane mobility. Total hip rotation showed the most pronounced change, accompanied by a very large effect size (d ≈ 1.5).

In contrast, changes in hip flexion were associated with a negligible effect size and thus were not considered clinically meaningful. No meaningful changes were observed in the non-operated hip for any ROM parameter.

**Figure 2:**
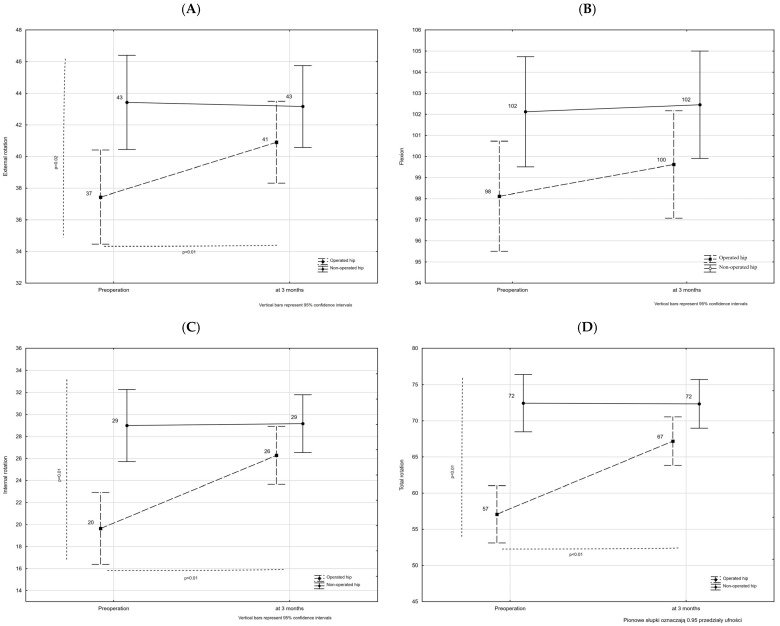
Changes in hip ROM between baseline and the 3-month follow-up in the operated and non-operated hips. (**A**) External rotation, (**B**) flexion, (**C**) internal rotation, and (**D**) total rotation.

Side-to-side differences in hip ROM at baseline and at the 3-month follow-up are illustrated in Figure 2. Before surgery, the operated hip demonstrated lower rotational ROM compared with the contralateral side. At the 3-month follow-up, a clear improvement was observed in the operated hip, particularly for external rotation, internal rotation, and total rotation, while the non-operated hip remained largely unchanged. Despite this improvement, measurable differences between the operated and non-operated hips persisted at the 3-month follow-up. In contrast, only minimal changes were observed in hip flexion over time.

### 3.4. Associations Between Hip ROM and HOOSs

Spearman rank correlation analysis demonstrated weak and inconsistent associations between hip active ROM and HOOS subscales at both time points, with correlation coefficients ranging from r = −0.34 to r = 0.31, and no consistent pattern observed. Notably, the highest observed correlation (external rotation vs. HOOS Total at 3 months; r = 0.31, *p* = 0.044) did not remain significant after correction for multiple comparisons.

In contrast, moderate correlations were identified at 3 months between the alpha angle measured in the anteroposterior projection and selected HOOS subscales, specifically Activities of Daily Living (ADL) (r = 0.41), Sport/Recreation (r = 0.38), Symptoms (r = 0.33), and Quality of Life (r = 0.32). No comparable associations were observed at baseline. After adjustment for multiple comparisons, only the association with the ADL subscale remained statistically significant.

## 4. Discussion

The results of this study partially support the initial hypothesis. FAIS is a complex clinical entity characterized by chronic pain and restricted hip mobility due to structural impingement. For persistent symptoms, arthroscopic intervention is the preferred treatment, aimed at repairing intra-articular lesions and restoring physiological kinematics through morphological correction. As predicted, our findings demonstrated clinically and statistically significant improvements across all HOOS subscales, accompanied by a substantial increase in active hip ROM—specifically internal rotation, external rotation, and total rotation—at the three-month follow-up.

However, contrary to our assumptions, only weak correlations were observed between these objective mobility measures and patient-reported outcomes. This finding suggests that perceived functional recovery in daily life may not be determined solely by the magnitude of restored joint mobility, highlighting the multifactorial nature of postoperative recovery. This recovery is likely facilitated not only by the restoration of joint mechanics but also by secondary factors such as the resolution of neuromuscular inhibition, decreased kinesiophobia, and the normalization of lumbopelvic motor control following the removal of the painful stimulus. Furthermore, psychosocial factors, including patient expectations, self-efficacy, and the psychological readiness to return to full activity, may significantly modulate the subjective perception of success, often independent of the objective clinical measures.

PROMs are the primary benchmarks for evaluating the short- and long-term efficacy of hip arthroscopy. The existing literature reflects a variety of outcome measures utilized across different follow-up periods [16,17]. Both Ramisetty et al. and Kemp et al. recommend a range of validated tools for assessing outcomes after hip arthroscopy, including the HOOS, which was the primary measure adopted in this study [4,18]. HOOS was selected due to its formal validation and cultural adaptation into the Polish language [15]. The current findings at the 3-month follow-up align with results reported by other authors, further supporting the early clinical benefits of arthroscopic intervention in this patient population [19,20]. Flores et al. also utilized the HOOS questionnaire and demonstrated that the most significant improvement occurred within the first 3 months postoperatively. In terms of mean scores, the participants in this study achieved higher improvements across all HOOS subscales compared to the data reported in the aforementioned study at the 3-month follow-up; this was particularly evident in the Pain (+27 vs. +20 points) and Sport (+34 vs. +21 points) domains. These differences could be attributed to variations in baseline characteristics, surgical techniques, or rehabilitation protocols, which may further limit direct comparability between cohorts.

The results regarding active ROM are consistent with a recent meta-analysis demonstrating postoperative improvements, particularly in rotational movements [8]. However, direct comparisons with existing literature are challenging, as the present study is among the first to specifically assess active ROM. Moreover, methodological heterogeneity across studies—including differences in testing positions, follow-up duration, and measurement tools—further limits comparability. The study by Freke et al. remains the most comparable to the present research, as both investigated post-hip arthroscopy patients using prone positions and inclinometry—a method similar to IMU-based measurements [9]. Notably, similar to the current study, the follow-up assessment of ROM was conducted 3 months postoperatively. While both studies observed ROM improvements, the present findings demonstrated a substantially greater increase in internal rotation (mean 7° vs. 1° in Freke et al.), reaching both statistical and clinical significance. This discrepancy may be attributed to the higher prevalence of cartilage damage in the cohort studied by Freke et al., a factor previously associated with restricted internal rotation in extension [21]. However, a notable difference concerns hip flexion, where Freke et al. reported a small but significant improvement that was not observed in the present cohort. Direct comparison is challenging due to the unique assessment protocol used in the present study. While Freke et al. assessed passive flexion in a supine position to a ‘hard end feel’, the standing active hip flexion test used in this study not only requires sufficient joint ROM but also adequate strength of the hip flexor muscles and appropriate pelvic mobility. These functional components may still be impaired three months after surgery, potentially masking mechanical joint gains when assessed in a weight-bearing, active manner [9,22].

Choi et al. also demonstrated a significant improvement in internal rotation (mean 10° vs. 7° in present study) [23]. The primary difference lay in the measurement methodology; the authors used a goniometer and assessed internal rotation at 90° of hip flexion. Therefore, the observed improvement may be more closely related to the surgical correction of the bony impingement. Interestingly, similar to the present findings, authors did not observe significant improvements in hip flexion at the 3-month follow-up. 

Despite a significant improvement in active ROM, deficits on the operated side relative to the contralateral limb persisted at the 3-month post-arthroscopy follow-up. However, these findings were comparable to those reported by other authors. For instance, in a study by Burlen et al., where rotation was assessed in the prone position 2.5 months after surgery, the side-to-side deficit relative to the contralateral side was 13% for internal rotation and 11.6% for external rotation (compared to 9.9% and 5.3%, respectively, in the present study) [5]. Notably, Tijsen et al. demonstrated that more than two years after hip arthroscopy, deficits of 10.7% for internal rotation (vs. 9.9% in present study), 7% for external rotation (vs. 5.3% in present study), and 3.3% for flexion persist, which corresponds with the current finding of a 2.8% flexion deficit at only 3 months [24]. These findings suggest that residual interlimb asymmetries may persist despite early postoperative improvements and should be considered when evaluating recovery trajectories following hip arthroscopy.

Interestingly, the present study demonstrated that improvements in active ROM occurred in movement directions that were not previously restricted by structural abnormalities such as CAM or pincer morphology. The most plausible explanation is a reduction in pain; however, the contribution of structural and mechanical changes at the level of the joint capsule, repaired labrum, and musculotendinous tissues cannot be excluded, particularly as these structures were subjected to stretching during postoperative rehabilitation [25,26]. This relationship may be bidirectional: while some motion gains may be driven by symptom resolution, persistent deficits relative to the contralateral side may reflect adhesions or ongoing healing processes within the aforementioned structures [26].

The results of the present study demonstrated predominantly weak associations between subjective outcomes and objective clinical measures. Only a limited number of studies in the literature have investigated these relationships in patients treated with hip arthroscopy, although most report findings similar to those of the current study [11,12]. In a study by Kemp et al., conducted on patients 12–24 months after hip arthroscopy, the authors examined the associations between PROMs and hip ROM. Similar to current findings, rotational ROM did not show meaningful correlations with patient-reported outcomes. However, in contrast to present results, a greater hip flexion ROM demonstrated moderate correlations with the HOOS-QOL and International Hip Outcome Tool-33 scores. One possible explanation for this discrepancy may be the methodological differences between the studies, including the passive assessment of ROM and the substantially longer follow-up period compared with the present study. A similar pattern, although observed in a cohort of patients with FAIS, has been reported in previous studies [27,28]. Gomes et al. demonstrated that an active hip flexion ROM exceeding 107°—rather than rotational ROM—was associated with a lower risk of severe symptoms. In another study, the authors reported a comparable relationship and, similarly to current findings, found no association with rotational ROM assessed in the prone position. Direct comparison of these results to the present findings regarding hip flexion is hindered by the aforementioned factors, specifically the active nature of the assessment and the standing, weight-bearing position used in this study.

Several limitations of this study should be acknowledged. First, the follow-up period was limited to three months, representing an early stage of postoperative recovery after hip arthroscopy. Although several studies have shown that the most substantial clinical improvement occurs within the first three months after surgery, further functional gains may continue for up to two years postoperatively [20]. Therefore, the relationships between objective measures and patient-reported outcomes observed in the present study may evolve over a longer follow-up period. In addition, the clinical evaluation was limited to a single PROM (the HOOS). This choice was dictated by the fact that, among the PROMs recommended for hip arthroscopy, only the HOOS has been formally validated for the Polish population. Future research should aim to include a broader battery of instruments, such as the iHOT-33, once validated versions become available in the local language. Second, the relatively small sample size may have limited the statistical power of the correlation analyses, particularly when examining subtle associations between objective and subjective measures. Third, the study did not include a control group, which restricts the ability to determine the extent to which the observed improvements were attributable solely to the surgical intervention rather than postoperative rehabilitation or natural recovery processes. Fourth, the study cohort included various concomitant procedures, such as labral repairs and microfractures (see Table 1). However, capsular closure was performed in all cases, serving as the primary determinant for postoperative ROM restrictions across the entire group. While microfractures required temporary weight-bearing modifications—which may have influenced subjective patient-reported outcomes—they did not alter the standardized range of motion progression. Finally, the ROM was assessed using active measurements. This approach may limit direct comparison with much of the existing literature, which predominantly relies on passive ROM. However, active testing may better reflect functional capacity in daily activities. It requires not only joint mobility, but also adequate muscle activation and neuromuscular control.

## 5. Conclusions

Hip arthroscopy for FAIS leads to significant early improvements in patient-reported outcomes and active ROM, particularly in rotational movements. However, measurable side-to-side deficits persist at three months postoperatively. The weak correlation between objective mobility gains and subjective functional scores suggests that patient-perceived recovery is multifactorial and not solely dependent on joint ROM. These results underline the importance of using objective tools, such as IMU sensors, to accurately monitor functional recovery beyond patient-reported symptoms. 

## Figures and Tables

**Table 1:** Baseline demographic and clinical characteristics of the study participants.

Variable	Study Group (*n* = 42)
Sex/men (%, n)	64 (27)
Mean ± SD	
Age (years)	36 ± 9
Weight (kg)	74 ± 14
Height (cm)	176 ± 8
BMI (kg/m^2^)	24 ± 3
LCEA (°)	35 ± 5
AA AP (°)	67± 13
AA Dunn (°)	61 ± 11
FAI morphology type(%, *n*)	
CAM	26 (11)
Pincer	12 (5)
Mixed(CAM and Pincer)	62 (26)
Concomitant procedure(%, *n*)	
Labrum repair	55 (23)
Labrum reconstruction	7 (3)
Labrum resection	21 (9)
Microfracture	24 (10)

Abbreviations: BMI = Body mass index; LCEA = Lateral Center Edge Angle; AA AP = Alpha Angle Anterior–Posterior View; AA Dunn = Alpha Angle Dunn View; FAI = Femoroacetabular Impingement.

**Table 2:** Changes in HOOSs between baseline and 3-month follow-up.

Variable	Preoperation	at 3 Months	*p*-Value	Cohen’s r
	Mean ± SD			
HOOS Total	57 ± 15	83 ± 11	<0.01	0.85
HOOS Symptoms	55 ± 19	77 ± 16	<0.01	0.68
HOOS Pain	59 ± 16	86 ± 11	<0.01	0.86
HOOS ADL	65 ± 18	91 ± 9	<0.01	0.83
HOOS Sport	44 ± 22	77 ± 16	<0.01	0.80
HOOS QOL	32 ± 14	57 ± 22	<0.01	0.76

Abbreviations: HOOS, Hip disability and Osteoarthritis Outcome Score; ADL, activities of daily living; QOL, quality of life; SD, standard deviation; r, effect size expressed as Cohen’s r.

**Table 3:** Changes in ROM between baseline and 3-month follow-up for operated and non-operated hips.

ROM (°)	Hip	Preoperation	at 3 Months	F	*p*-Value (Interaction)
		Mean ± SD			
Flexion	Operated	98 ± 9	100 ± 8	1.90	0.17
	Non-operated	102 ± 8	103 ± 8		
External rotation	Operated	37 ± 10	41 ± 8	11.55	0.01
	Non-operated	43 ± 9	43 ± 8		
Internal rotation	Operated	20 ± 12	26 ± 9	36.10	<0.01
	Non-operated	29 ± 9	29 ± 9		
Total rotation	Operated	57 ± 15	67± 12	60.09	<0.01
	Non-operated	72 ± 11	72 ± 10		

Note. Values are presented as mean ± SD. ROM, range of motion; SD, standard deviation; F, F-statistic (analysis of variance); p, probability value. F and *p*-values represent the interaction effect (time × side) from a two-way repeated-measures ANOVA (2 × 2 design).

## Data Availability

The datasets generated and/or analyzed during the current study are not publicly available due to privacy/ethical restrictions but are available from the corresponding author upon reasonable request.
